# Agreement and Reliability Between Urine Reagent Strips and Refractometry for Field Assessment of Hydration in Ultra-Trail Runners

**DOI:** 10.3390/nu18030466

**Published:** 2026-01-31

**Authors:** Daniel Rojas-Valverde, Volker Scheer, Marcelo Tuesta, Carlos D. Gómez-Carmona

**Affiliations:** 1 Centro de Investigación, Desarrollo e Innovación en Salud y Deporte (CIDISAD), Escuela Ciencias del Movimiento Humano y Calidad de Vida (CIEMHCAVI), Universidad Nacional, Heredia 86-3000, Costa Rica; drojasv@una.cr; 2 Clínica de Lesiones Deportivas (Rehab & Readapt), Escuela Ciencias del Movimiento Humano y Calidad de Vida (CIEMHCAVI), Universidad Nacional, Heredia 86-3000, Costa Rica; 3 Ultra-Sports Science Foundation, 69310 Pierre-Bénite, France; volkerscheer@yahoo.com; 4 Exercise and Rehabilitation Sciences Laboratory, School of Physical Therapy, Faculty of Rehabilitation Sciences, Universidad Andres Bello, Santiago 7550000, Chile; marcelo.tuesta@unab.cl; 5 Laboratory of Sport Sciences, Centro de Medicina Deportiva Sports MD, Viña del Mar 2520000, Chile; 6 Research Group in Training, Physical Activity and Sports Performance (ENFYRED), University of Zaragoza, 44003 Teruel, Spain; 7 Research Group in Optimization of Training and Sports Performance (GOERD), Faculty of Sport Science, University of Extremadura, 10005 Caceres, Spain; 8 BioVetMed & SportSci Research Group, University of Murcia, 30100 Murcia, Spain

**Keywords:** hydration, dehydration, physical performance, sports medicine, runners, urine-specific gravity

## Abstract

**Background/Objectives**: Accurate hydration assessment is critical for optimizing performance and preventing heat-related complications in ultra-endurance athletes. This study evaluated the agreement and reliability between urine reagent strips and refractometry for field-based hydration assessment via urine-specific gravity (USG) in ultra-trail runners. **Methods**: Thirty-four ultra-trail runners (22 males, 12 females; mean age 43.71 ± 11.50 years) participated during The Coastal Challenge, a 241-km multi-stage ultra-trail competition. Urine samples were collected before and after the first two stages (Stage 1: 41 km, 1071 m elevation; Stage 2: 40 km, 1828 m elevation). USG was measured using semi-quantitative urine reagent strips (Combur10Test M) and a handheld digital refractometer (Palm Abbe™). Agreement was assessed via paired *t*-tests, Pearson and Spearman correlations, intraclass correlation coefficients, and Bland-Altman plots across four measurement time points. **Results**: Strong agreement existed between methods with correlation coefficients of 0.92–0.99 (*p* < 0.01) within the hydration range typical of well-prepared ultra-endurance athletes (USG 1.010–1.020). No significant differences were found between devices at any time point (all *p* > 0.05). Bland-Altman analyses revealed minimal mean bias (range: −0.002 to +0.001 g/mL) and narrow limits of agreement, with fewer than 5% of values falling outside limits. Both methods detected significant increases in USG from pre- to post-stage (*p* < 0.01), indicating exercise-induced hypohydration. **Conclusions**: Semi-quantitative urine reagent strips and handheld refractometers demonstrate strong agreement for hydration assessment in ultra-trail runners under field conditions when not severely hypohydrated, supporting their interchangeable use for practical monitoring.

## 1. Introduction

The global popularity of ultra-trail running has experienced significant growth in recent decades [1]. It is driven by interest in outdoor physical activity, personal achievement, and lifestyle-related health promotion [2]. Participation in long-distance trail events has increased across amateur, youth, master’s and professional populations [3,4,5]. These competitions demand prolonged physical effort over uneven terrain and variable climatic conditions in challenging environments. Multi-stage races present unique physiological demands that require close monitoring, as cumulative fatigue and environmental exposure can compound health risks [6,7].

Ultra-trail runners are exposed to various physiological stressors that may predispose them to acute and chronic health issues [8,9,10]. Common concerns include musculoskeletal injuries, gastrointestinal distress, thermoregulatory strain, and fluid-electrolyte imbalances [6]. Among these, hypohydration and dehydration remain one of the most frequent and potentially dangerous conditions, impairing cardiovascular function, thermoregulation, and performance. Early identification of suboptimal hydration status is therefore critical to maintaining health and performance during prolonged endurance events (e.g., multistage events) [11,12,13].

Hydration status can be assessed through various methods. Laboratory measures such as plasma and urine osmolality are considered gold standards but are often impractical in field conditions [14,15]. Refractometry of urine-specific gravity (USG) is widely regarded as the practical gold standard due to its accuracy, portability, and simplicity [16,17]. USG provides a reliable indicator of urine concentration and, by extension, hydration status, as more concentrated urine typically reflects reduced body water or increased solute load [16,17]. It reliably reflects urine concentration and is commonly used to validate other field methods, such as dipsticks and urine colour charts. Body weight changes, bioelectrical impedance, heart rate, and perceptual measures are also employed in applied settings to provide a comprehensive view of hydration status [18]. Among field-practical options, urine reagent strips offer a cost-effective alternative for USG assessment, though their agreement with refractometry requires validation in competitive endurance environments.

Previous research examining the validity and agreement between urine reagent strips and refractometers has yielded mixed results. Abbey et al. [19] demonstrated strong agreement in controlled laboratory settings, whereas Adams et al. [20] questioned their field applicability after finding strips inaccurate for detecting hypohydration. Variable concordance has been documented across laboratory and team sports contexts [21,22,23], though these validation studies were primarily conducted under controlled conditions or in shorter-duration activities. Ultra-trail running, however, presents distinct challenges that may affect both hydration status and measurement accuracy, including prolonged exercise duration (often 6–12 h per stage), cumulative multi-day physiological stress, and highly variable environmental conditions [6,7,11]. Therefore, the present study evaluated the agreement and reliability between a handheld refractometer and urine reagent strips in assessing USG in ultra-trail runners during a two-day, multi-stage competition. By comparing these two commonly used field methods across pre- and post-race measurements, this study sought to determine the suitability and interchangeability of these tools for monitoring hydration status in real-world endurance sports settings.

## 2. Materials and Methods

### 2.1. Design

**Figure 1:**
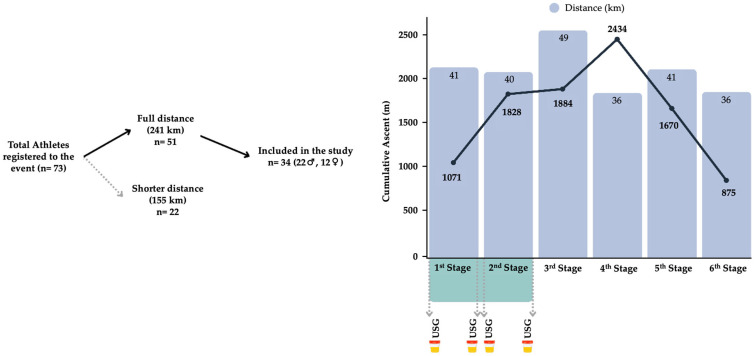
Runners’ recruitment process and schematic design of the study.

This prospective observational study was conducted during a 241 km (9762 positive meters of elevation gain) multistage ultra-trail running event (The Coastal Challenge). Based on the study’s aim, only the first two stages were included in the analysis. Urine samples were collected at four time points, both before and after each race stage, and analysed immediately by researchers under standardised conditions. Stage 1 covered 41 km with 1071 m of elevation gain, while Stage 2 comprised 40 km with 1828 m of ascent (see Figure 1). Both stages were completed on consecutive days, starting at 5 am and finishing around 2 pm. Environmental conditions showed mean temperature and humidity at start of ~22 °C and ~90%, and at finish of ~30 °C and ~65%, with no significant differences between stages (*p* > 0.05). Liquid intake and feeding were ad libitum.

### 2.2. Participants

Sample size was determined a priori using G*Power 3.1.9.7 (Heinrich-Heine-Universität Düsseldorf, Düsseldorf, Germany) [24]. For correlation analyses with an expected effect size of r = 0.90, α = 0.05, and statistical power of 0.95, a minimum of 29 participants was required. A total of 34 ultra-trail runners (22 males, 12 females) volunteered to participate in this study, providing 136 paired measurements across four time points, which adequately powered the study to detect strong agreement between methods. Participants were recruited during the pre-race meeting and were included based on their registration and active participation in the full-distance race.

Participants’ characteristics included a mean age of 43.71 (11.50) years, body mass of 81.11 (36.98) kg and height of 1.68 (0.32) m. All runners were experienced in endurance trail running, with the majority (*n* = 26, 76.5%) having over five years of trail running experience and all having completed at least one ultra-trail competition (more than 50 km) in the previous year. Most participants identified as competitive-level athletes (*n* = 18, 52.9%) or recreational-level athletes (*n* = 16, 47.1%), and were from 10 different nationalities, reflecting a diverse and well-trained cohort.

Participants were informed about the study’s aims and procedures. They provided written informed consent one day before the event, during official race registration, via a digital consent form. The study protocol was approved by the corresponding institutional ethics committee and adhered to the principles outlined in the Declaration of Helsinki. The protocol was approved by the Institutional Review Boards of the Universidad Nacional (Reg. Code 2019-P005) and University of Extremadura (Reg. Code 139/2020).

### 2.3. Urine Collection and Analysis

Urine samples were collected on-site immediately before and after each stage using sterile 30 mL polypropylene containers (Nipro Medical Corp., Osaka, Japan). Samples were analysed using two methods: urine reagent strips (Combur10Test M, Roche, Mannheim, Germany) [10,25] and a handheld digital refractometer (Palm Abbe™, Misco, Solon, OH, USA) [22,26]. Two independent health technicians interpreted the strip results using the manufacturer’s colour scale, with discrepancies resolved by a third evaluator. The USG was assessed using a refractometer after calibration and cleaning. Hydration status based on USG was classified according to established reference ranges [27]: euhydrated (USG < 1.010), minimal hypohydration (1.010–1.020), significant hypohydration (1.021–1.030), or serious hypohydration (>1.030). Urine collection was standardised to occur at the same hour immediately before and after each stage to minimise diurnal variation in urine concentration. No participants reported discomfort or urination issues during the collection process.

### 2.4. Statistical Analysis

A statistical analysis was conducted to assess the agreement and reliability between two methods of measuring urine-specific gravity: a manual refractometer (“Ref”) and colorimetric urine reagent strips (“Strip”). The analysis included measurements taken before and after Stages 1 and 2 of the multi-stage ultra-race. For each measurement pair (PreS1Ref vs. PreS1Strip, PostS1Ref vs. PostS1Strip, PreS2Ref vs. PreS2Strip, and PostS2Ref vs. PostS2Strip), the mean and standard deviation (*SD*) were calculated. The mean difference (∆ change) between the two methods was computed, and a paired Student’s t-test was performed to determine if significant differences existed between the methods. For this analysis, Strip USG values were treated as continuous variables. The Intraclass Correlation Coefficient (ICC) was calculated using the ICC_(2,1)_ model, which corresponds to a two-way mixed-effects model for single measures, assessing consistency. The *ICC* was derived from the variance components (between-subjects, between-methods, and residual) using mean squares obtained through ANOVA decomposition. To assess the relationship between methods, Pearson’s (*r*) and Spearman’s rank (*r_s_*) correlation coefficients were computed, with the latter accounting for the ordinal nature of urine reagent strip data.

Agreement between the two measurement techniques was further evaluated using Bland–Altman plots [28], which visualise the mean difference and limits of agreement (±1.96 SD). These plots allow for the assessment of systematic bias and the dispersion of differences across the measurement range. The figures were generated using the DATAtab online statistical calculator (DATAtab e.U., Graz, Austria). A summary table was generated including mean (SD), ∆ change, *t*-statistic, *p*-value, ICC, Pearson’s *r*, and its significance level for each measurement comparison. Statistical significance was set at *p* < 0.05. All analyses were conducted using SPSS v27 (IBM, Chicago, IL, USA).

## 3. Results

Participants completed stage 1 in an average time of 6 h, 3 min, and 34 s (*SD* = 1 h, 18 min, and 50 s), and stage 2 in 5 h, 21 min, and 14 s (*SD* = 2 h, 7 min, and 45 s). USG increased significantly comparing pre vs. post measurements in stages 1 and 2 when comparing pre vs. post measurements in both equipment: Strips Stage 1 (*t* = 3.4, *p* < 0.01) and stage 2 (*t* = 3.5, *p* < 0.01), refractometer stage 1 (*t* = 1.04, *p* < 0.01) and stage 2 (*t* = 2.55, *p* < 0.01). On average, USG values indicated minimal hypohydration (USG 1.010–1.020) according to the classification criteria established in the methods (Section 2.3) [27].

**Figure 2:**
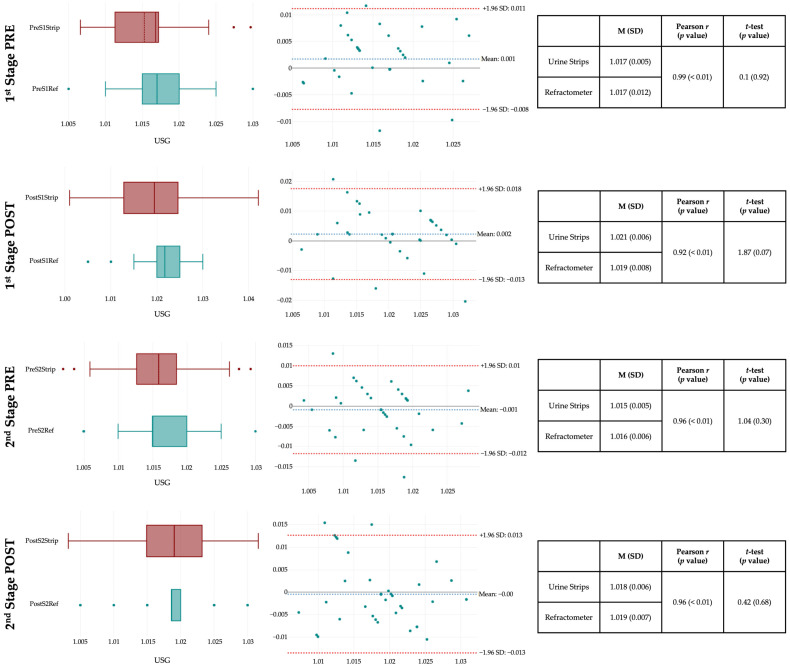
Agreement and reliability of urine-specific gravity assessed by refractometer vs. urine strips. **Note.** The right panels show box-and-whisker plots for each measurement time point, where the central band represents the median, the box boundaries indicate the 25th and 75th percentiles, and the whiskers extend to the minimum and maximum values. The middle panels display Bland-Altman plots, where the *x*-axis represents the mean of the two methods [(Refractometer + Strip)/2], and the *y*-axis represents the difference between methods (Refractometer − Strip). The solid black horizontal line at 0 indicates perfect agreement, the blue dotted line labelled “Mean” represents the mean difference between methods (bias), and the red dotted lines represent the limits of agreement (mean difference ± 1.96 SD).

Urine-specific gravity measurements obtained via urine strips and a handheld refractometer showed strong agreement and reliability across both exercise stages (see Figure 2). Both Pearson (*r* = 0.92 to 0.99) and Spearman rank (*r_s_* = 0.91 to 0.98) correlation coefficients were nearly identical and highly significant (all *p* < 0.01), indicating excellent agreement between methods and demonstrating that the ordinal nature of strip data did not affect the strength of the relationship. No statistically significant differences were found between the two techniques in any condition (all *p* > 0.05), and Bland-Altman plots demonstrated narrow limits of agreement with minimal bias, particularly in pre-exercise assessments. Less than 5% of the values fell outside these limits, and no pattern of heteroscedasticity was observed. Slightly greater variability was observed post-exercise, potentially due to physiological changes in urine concentration. Overall, both methods proved consistent and comparable, with the refractometer showing slightly tighter distributions, which may make it marginally more sensitive for detecting subtle changes in urine concentration.

## 4. Discussion

The present study investigated the agreement and reliability between a handheld refractometer and urine reagent strips for assessing urine-specific gravity (USG) in ultra-trail runners during a multistage competition. A high degree of concordance was found between the two measurement methods at pre- and post-exercise conditions in the two race stages. The correlation coefficients were consistently strong (*r* = 0.92–0.99), and no statistically significant differences were found between methods at any time point. Bland-Altman analyses revealed narrow limits of agreement and minimal bias, particularly under resting conditions, indicating that both tools yield comparable USG-based hydration status assessments.

The findings obtained in this study extend previous validation studies by demonstrating strong agreement between methods in ultra-trail runners during competition. Abbey et al. [19] reported good validity between methods in controlled laboratory conditions. Our results indicated excellent concordance under ultra-endurance competition conditions across USG values in the minimal hypohydration range (USG = 1.010–1.020). This range is consistent with hydration states in well-prepared ultra-endurance athletes who maintain adequate fluid intake [29]. Post-exercise measurements showed a slight increase in variability, which may be attributed to acute exercise-induced changes in urine osmolality, pH, and the presence of interfering solutes that can influence the colorimetric reaction of urine strips [30]. Despite this, overall reliability remained within acceptable limits, supporting the field applicability of both instruments for USG-based hydration monitoring during training, pre-competition, and recovery.

However, our findings differ from those reported by Adams et al., who questioned the field applicability of reagent strips after finding them inaccurate for detecting hypohydration [20]. The discrepancy between studies could be attributed to several factors, including differences in hydration status, sport modality, environmental conditions [21,22,23,25], and importantly, the use of different reagent strip brands. Adams et al. [20] used Siemens Multistix 10 SG reagent strips, whereas the current study used Combur10Test M strips by Roche. These different brands may have varying colorimetric formulations, USG detection ranges, and reading sensitivities, which could influence measurement accuracy and agreement with refractometry. Additionally, urine reagent strip performance at USG values >1.030, indicating severe hypohydration, warrants further investigation as increased solute concentration may affect colorimetric accuracy [18,31].

Moreover, the observed increase in USG post-stage, particularly in Stage 2, is consistent with exercise-induced hypohydration. Although USG values remained within clinically acceptable thresholds, these findings underscore the physiological demands imposed by prolonged endurance activity and the importance of adequate fluid replacement strategies in ultra-endurance settings [32,33]. Measurements during the first two stages captured acute hydration responses typical of early race segments, consistent with research showing significant dehydration manifests within the first 40–50 km [34]. The ad libitum intake approach reflects self-regulated patterns typical in ultra-endurance competition, aligning with current individualized hydration recommendations [27,35]. Importantly, while USG is a validated hydration biomarker, definitive quantification of fluid deficits would require body mass measurements alongside USG assessments [14,15]. Nevertheless, the aim to evaluate method agreement was fully addressed as both methods demonstrated excellent concordance, providing evidence for their practical interchangeability in ultra-endurance settings where minimal to moderate hypohydration commonly occurs.

From a practical standpoint, urine reagent strips offer significant advantages for field-based hydration monitoring in ultra-endurance settings. Their portability, low cost, and ease of use make them accessible to athletes, support crews, and medical staff [27,36]. However, proper interpretation requires adequate lighting conditions and accurate color chart comparison, which may be challenging for fatigued athletes during competition. Color perception can be affected by environmental factors such as artificial lighting, shadows, and individual visual differences, potentially compromising accuracy [18]. Therefore, strips are best utilized during pre-race assessment, at aid stations with support personnel, or post-race recovery monitoring rather than self-assessment mid-competition. Refractometers, while providing higher precision, require more technical expertise and calibration but eliminate subjective color interpretation [14].

Beyond single-indicator approaches, the WUT (Weight, Urine, Thirst) framework proposed by Cheuvront and Kenefick [37] provides a comprehensive field assessment strategy that integrates body weight changes, urine indices, and thirst perception. Recent validation studies have demonstrated strong relationships between WUT criteria and gold-standard hydration biomarkers [38,39,40], supporting its utility for practical hydration monitoring in athletic populations. In ultra-trail running contexts, where laboratory methods are impractical, this multi-component approach offers advantages. Body weight monitoring at aid stations can be combined with urine assessment using either strips or refractometry, as validated in our findings, alongside continuous athlete self-monitoring of thirst perception—a practical component requiring no equipment [41]. This integrated strategy acknowledges that no single indicator perfectly reflects hydration status, but together they provide complementary information for individualized fluid intake decisions during prolonged endurance events.

In summary, this study confirms that both urine strips and refractometers are valid tools for USG-based hydration status in ultra-endurance athletes, with strips offering logistical advantages and refractometers providing slightly higher precision under elevated physiological stress. However, limitations include the relatively small sample size, specific ultra-trail running population characteristics that may limit generalizability, data collection limited to the first two stages, and the narrow USG range observed (euhydrated to minimal hypohydration), which reduced variability for robust reliability estimates. Although fluid and food intake were ad libitum during race stages, individual consumption data were not systematically recorded, which may have influenced USG measurements; however, this approach reflects authentic field conditions and enhances the ecological validity of our findings. Additionally, urine reagent strips provide semi-quantitative results and may be influenced by variables unrelated to hydration status, such as urine pH or solutes like glucose or proteins. Future research should investigate these tools’ validity across broader hydration ranges, examine agreement throughout entire multi-stage events, and validate findings in larger and more diverse samples across varying environmental conditions. Assessing their predictive validity for performance outcomes and hydration-related health risks would further strengthen the evidence base for field-based hydration monitoring in endurance sports.

## 5. Conclusions

This study demonstrated strong agreement and reliability between handheld refractometers and urine reagent strips in measuring USG among ultra-trail runners across two race stages. Both methods detected significant increases in USG indicating minimal hypohydration, particularly in Stage 2, despite similar completion times and environmental conditions between stages. Runners maintaining ad libitum fluid intake showed predominantly minimal hypohydration with no cases of decreased USG, suggesting adequate self-regulation of fluid intake.

While both tools are suitable for field use, urine strips offer a particularly viable and cost-effective screening tool for resource-limited field settings, making hydration monitoring accessible to support crews and medical staff without requiring specialized equipment, whereas refractometers provide slightly greater precision, particularly post-exercise. These findings support the use of either method for routine USG-based hydration monitoring in ultra-endurance settings when not severely hypohydrated, with strips offering a practical alternative when resources are limited. USG provides the advantage of being non-invasive, rapidly obtainable in field conditions, and less affected by acute plasma volume shifts. However, USG assessment should be integrated within comprehensive hydration evaluation alongside body mass changes and thirst perception (as proposed by the WUT framework), complementing athlete-directed fluid intake strategies during competition.

## Data Availability

The data presented in this study are available on request from the corresponding author. The data are not publicly available due to privacy concerns.
